# Video text messaging is needed to deliver patient education about preventive care in the United States

**DOI:** 10.1371/journal.pdig.0000258

**Published:** 2023-05-30

**Authors:** Gloria D. Coronado, Esmeralda Ruiz, Evelyn Torres-Ozadali, Jamie H. Thompson, Jennifer S. Rivelli, Annie Thibault, Anne L. Escaron

**Affiliations:** 1 Center for Health Research, Kaiser Permanente Northwest, Portland, Oregon, United States of America; 2 Institute for Health Equity, AltaMed Health Services Corporation, Los Angeles, California, United States of America; 3 Colorectal Cancer Prevention Network, University of South Carolina, Columbia, South Carolina, United States of America; Iran University of Medical Sciences, IRAN (ISLAMIC REPUBLIC OF)

The penetration of smart phones and apps has revolutionized modern life, from education to scientific data collection to interpersonal communication. One area of contemporary life where smart phone use has failed to live up to its revolutionary potential, however, is in health-information messaging. Despite the very high penetration of smart phones—97% of people in the United States now own a cell phone of some type—most health care systems are not taking full advantage of cell phones to deliver health messages. [1] Those that do rely primarily on texting or calling (a practice that was further spurred by the COVID-19 pandemic), and not on linked video messaging. [2–4]

While text messaging can reach far more people than in-person educational sessions or live phone calls, for a fraction of the cost, text messaging alone has limitations. The conversion rate for getting people to complete a screening test, for example, is higher with a live call than with texting alone. [3,5] This lower conversion rate is one of many reasons to expand the use of health text messaging to include a video component. Video messaging can help overcome language and health-literacy barriers, is more engaging, and can demonstrate how to do a task that might be difficult to explain solely using words.

Messaging directly to patients’ cell phones shifts away from relying on clinic-specific patient portals that are unequally used across population subgroups. [6] This inequity of access to health care and to patient portals is one of the reasons that the Centers for Medicaid and Medicare (CMS) is driving efforts to use app-based health information portals rather than relying solely on EMR- or web-based health portals. The rule CMS released in October 2022 stated specifically that a major aim of the new set of data standards is to create “processes …to help ensure that patients remain at the center of their own care.” [7] Cell-phone based apps that use video could help health systems meet this goal.

One example to look to is a program taking place at AltaMed Health Services Corporation, which is a large urban federally qualified health center that operates clinics in Los Angeles and Orange Counties in California. The program sends short (<1 min) animated videos for both English- and Spanish-speakers about the importance of colorectal cancer (CRC) screening and instructs patients on how to complete a fecal immunochemical test (FIT). The videos address common errors in FIT completion (e.g., too much/too little sample collected, collection date omitted; videos available on the project website. [8]) The videos address health messages about routine screening for average-risk adults and can be sent without privacy concerns. Clinic staff incorporated these videos into a multi-component intervention that uses text messages to boost participation in CRC screening among patients aged 45–75. AltaMed serves nearly 250,000 patients each year, 75,000 of whom are aged 45–75.

Screening rates of all races are also dramatically low among adults aged 45–49, who became eligible for screening in May 2021 (only 1.4% of eligible 45-49-year-olds had completed CRC screening at AltaMed Health Services Corporation). This is troubling given that CRC is 90% curable with timely detection and appropriate treatment of precancerous polyps, and increased screening could reduce CRC incidence by up to 50%. [9] Over 80% of adults who receive care at AltaMed are Latinx ethnicity. Compared to younger Latinx adults, Latinx adults who are age-eligible for CRC screening tend to have less English fluency, worse access to the internet, and lower health literacy, making them ideal candidates for video messaging. [10]

Between February and July 2021, AltaMed’s centralized outreach staff sent an introductory text message to a list of patients due for CRC screening asking whether the patient would like to be mailed a FIT test to screen for CRC (opt in). Patients who opted in (n = 2,509) were mailed a FIT and texted a link to an animated educational video (Fig 1). Patients who did not return their FIT after a month were sent a text reminder and delivered a live phone call from a health center outreach staff, as needed.

**Fig 1:**
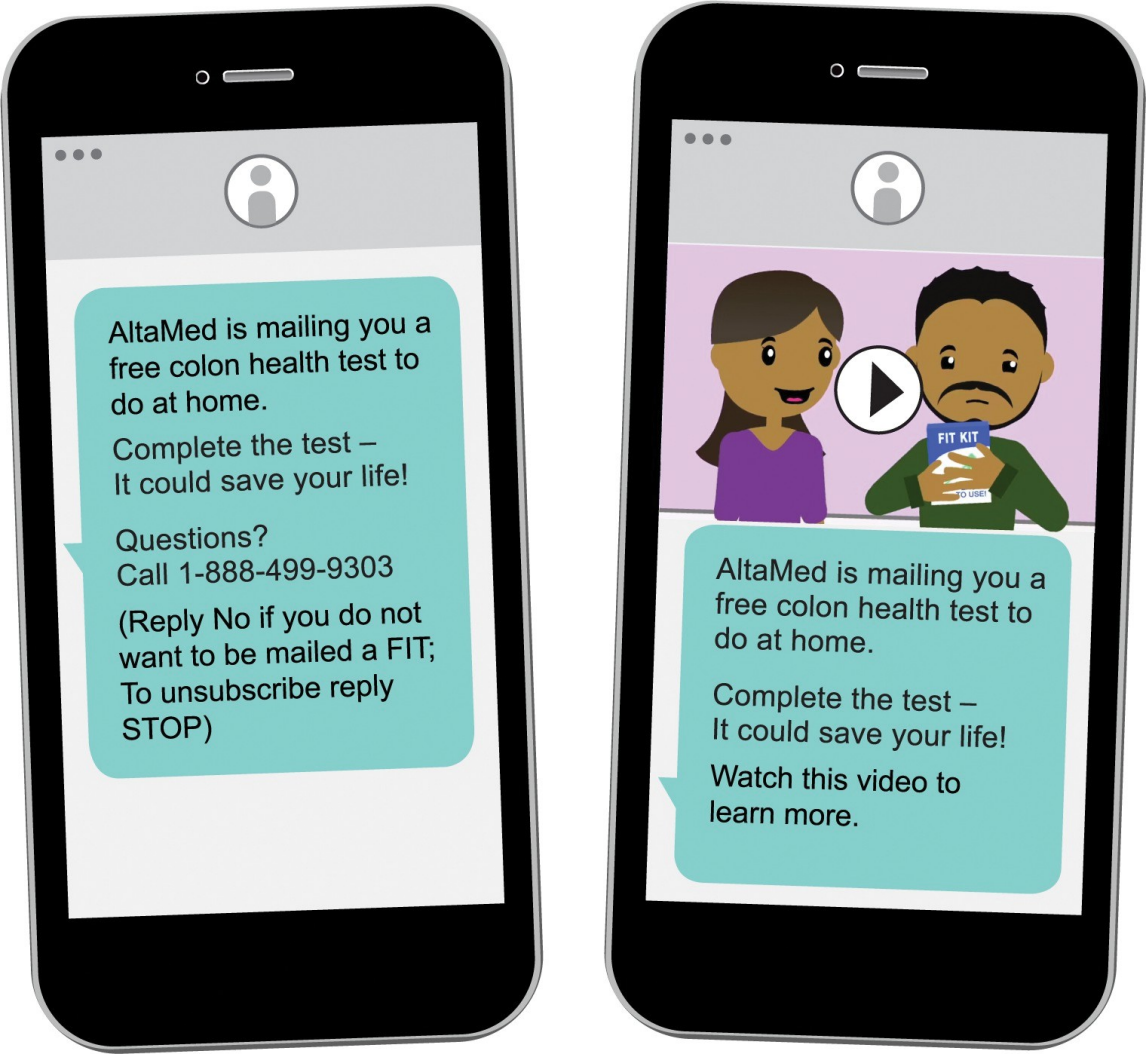
Image of Standard Text Message and Video Text Message.

The program yielded a 40% FIT return rate, nearly double the average return rate reported in systematic reviews of mailed FIT outreach. [11–13] Feedback to date suggests that the program is popular with patients as well as with the health professionals delivering the program. One health professional noted: “… patients that click on the link really like the video and it has helped them properly complete the test.” Patients reported sharing the video with friends and family members. Importantly, health care professionals also noted that the program is sustainable without any additional funding beyond standard text messaging fees, which average $0.04 per text message.

In future work we plan to obtain direct feedback from patients and health care professionals about how to improve the program and conduct a robust evaluation of the program to test its effectiveness overall and among population subgroups, including patients whose language preference is English vs. Spanish, and among patients with and without prior CRC screening history. This could allow message content to be tailored to patients’ preferred language, and personal experience with screening.

Video-texting is a low-cost, digital solution that can be used by clinics to educate community members they serve about evidence-based preventive health services. Previous research has delivered text message reminders or used videos in clinic waiting rooms or exam rooms. [3,4,14] The current program uniquely combines these two approaches as a resource-efficient strategy to increase awareness about CRC and the need for screening.

More broadly, video text messaging delivered by trusted sources may prove useful to counter misinformation and myths about any health topic. Disseminating accurate health information can support healthful lifestyle behaviors and appropriate and timely care practices, improve decision-making about health, build resilience to misinformation, and restore public trust in evidence-based medicine. Now is the time for health care systems in the United States (and in other countries where smartphone ownership rates are high) to make use of cell phones to deliver health messages.

## References

[1] Center. PR. Mobile Fact Sheet 2021 [3/3/2023]. Available from: https://www.pewresearch.org/internet/fact-sheet/mobile/.

[2] Huf SW, Asch DA, Volpp KG, Reitz C, Mehta SJ. Text Messaging and Opt-out Mailed Outreach in Colorectal Cancer Screening: a Randomized Clinical Trial. Journal of general internal medicine. 2021;36(7):1958–64. Epub 2021/01/30. doi: 10.1007/s11606-020-06415-8 ; PubMed Central PMCID: PMC8298623. 33511567 PMC8298623

[3] Coronado GD, Rivelli JS, Fuoco MJ, Vollmer WM, Petrik AF, Keast E, et al. Effect of Reminding Patients to Complete Fecal Immunochemical Testing: A Comparative Effectiveness Study of Automated and Live Approaches. Journal of general internal medicine. 2018;33(1):72–8. Epub 2017/10/12. doi: 10.1007/s11606-017-4184-x ; PubMed Central PMCID: PMC5756165. 29019046 PMC5756165

[4] Coronado GD, Nyongesa DB, Petrik AF, Thompson JH, Escaron AL, Younger B, et al. Randomized Controlled Trial of Advance Notification Phone Calls vs Text Messages Prior to Mailed Fecal Test Outreach. Clin Gastroenterol Hepatol. 2020. Epub 2020/08/03. doi: 10.1016/j.cgh.2020.07.053 . 32739569 PMC9285860

[5] Coronado GD, Nyongesa DB, Petrik AF, Thompson JH, Escaron AL, Younger B, et al. Randomized Controlled Trial of Advance Notification Phone Calls vs Text Messages Prior to Mailed Fecal Test Outreach. Clinical gastroenterology and hepatology: the official clinical practice journal of the American Gastroenterological Association. 2021;19(11):2353–60.e2. Epub 20200730. doi: 10.1016/j.cgh.2020.07.053 ; PubMed Central PMCID: PMC9285860. 32739569 PMC9285860

[6] Perrin A TE. Smartphones help blacks, Hispanics bridge some–but not all–digital gaps with whites. 2021 [cited 2022 July 13]. Available from: https://www.pewresearch.org/fact-tank/2019/08/20/smartphones-help-blacks-hispanics-bridge-some-but-not-all-digital-gaps-with-whites/.

[7] Services CfMM. CMS Interoperability and Patient Access Final Rule July 1, 2021. Available from: https://www.cms.gov/regulations-and-guidance/guidance/interoperability/index#CMS-Interoperability-and-Patient-Access-Final-Rule.

[8] Coronado GD. Mailed FIT outreach to improve CRC screening 2022 [cited 2022 March 26]. Available from: www.mailedFIT.org.

[9] Meester RG, Doubeni CA, Lansdorp-Vogelaar I, Goede SL, Levin TR, Quinn VP, et al. Colorectal cancer deaths attributable to nonuse of screening in the United States. Ann Epidemiol. 2015;25(3):208–13.e1. Epub 2015/02/28. doi: 10.1016/j.annepidem.2014.11.011 ; PubMed Central PMCID: PMC4554530. 25721748 PMC4554530

[10] Tappen RM, Cooley ME, Luckmann R, Panday S. Digital Health Information Disparities in Older Adults: a Mixed Methods Study. J Racial Ethn Health Disparities. 2022;9(1):82–92. Epub 20210107. doi: 10.1007/s40615-020-00931-3 ; PubMed Central PMCID: PMC7790471. 33415705 PMC7790471

[11] Dougherty MK, Brenner AT, Crockett SD, Gupta S, Wheeler SB, Coker-Schwimmer M, et al. Evaluation of Interventions Intended to Increase Colorectal Cancer Screening Rates in the United States: A Systematic Review and Meta-analysis. JAMA internal medicine. 2018;178(12):1645–58. Epub 2018/10/17. doi: 10.1001/jamainternmed.2018.4637 . 30326005 PMC6583619

[12] Jager M, Demb J, Asghar A, Selby K, Mello EM, Heskett KM, et al. Mailed Outreach Is Superior to Usual Care Alone for Colorectal Cancer Screening in the USA: A Systematic Review and Meta-analysis. Dig Dis Sci. 2019;64(9):2489–96. doi: 10.1007/s10620-019-05587-6 Epub 2019 Mar 26. 30915656 PMC6706307

[13] Issaka RB, Avila P, Whitaker E, Bent S, Somsouk M. Population health interventions to improve colorectal cancer screening by fecal immunochemical tests: A systematic review. Preventive medicine. 2019;118:113–21. Epub 2018/10/28. doi: 10.1016/j.ypmed.2018.10.021 ; PubMed Central PMCID: PMC6322951. 30367972 PMC6322951

[14] Berkhout C, Zgorska-Meynard-Moussa S, Willefert-Bouche A, Favre J, Peremans L, Van Royen P. Audiovisual aids in primary healthcare settings’ waiting rooms. A systematic review. Eur J Gen Pract. 2018;24(1):202–10. doi: 10.1080/13814788.2018.1491964 ; PubMed Central PMCID: PMC6104610. 30132369 PMC6104610

